# Dietary factors and risk for asthma: A Mendelian randomization analysis

**DOI:** 10.3389/fimmu.2023.1126457

**Published:** 2023-02-22

**Authors:** Wenwen Yang, Yanjiang Yang, Li He, Min Zhang, Shuo Sun, Feng Wang, Biao Han

**Affiliations:** ^1^ The First Clinical Medical College, Lanzhou University, Lanzhou, Gansu, China; ^2^ Qilu hospital of Shandong University, Shandong University, Jinan, Shandong, China; ^3^ Department of Thoracic Surgery, the First Hospital of Lanzhou University, Lanzhou, Gansu, China; ^4^ Gansu Province International Cooperation Base for Research and Application of Key Technology of Thoracic Surgery, The First Hospital of Lanzhou University, Lanzhou, Gansu, China

**Keywords:** Mendelian randomization, dietary intake, asthma, alcohol intake, fruit intake

## Abstract

**Background:**

Previous research has found a link between dietary factors and asthma. However, few studies have analyzed the relationship between dietary factors and asthma using Mendelian randomization. Methods: The IEU Open GWAS project (https://gwas.mrcieu.ac.uk/) was the source of exposure and outcome datasets. The exposure datasets included Alcoholic drinks per week, Alcohol intake frequency, Processed meat intake, Poultry intake, Beef intake, Non-oily fish intake, Oily fish intake, Pork intake, Lamb/mutton intake, Bread intake, Cheese intake, Cooked vegetable intake, Tea intake, Fresh fruit intake, Cereal intake, Salad/raw vegetable intake, Coffee intake, and Dried fruit intake. The weighted median, MR-Egger, and Inverse Variance Weighted methods were used as the main methods of Mendelian randomization analysis. Heterogeneity and pleiotropic analysis were performed to ensure the accuracy of the results.

**Results:**

Alcohol intake frequency (after removing outliers OR: 1.217; 95% CI: 1.048-1.413; p=0.00993) was related to an increased risk of Asthma. Fresh fruit intake (OR: 0.489; 95% CI: 0.320-0.748; p=0.000954) and Dried fruit intake (after removing outliers OR: 0.482; 95% CI: 0.325-0.717; p= 0.000312) were discovered as protective factors. Other dietary intakes found no causal relationship with asthma.

**Conclusion:**

This study found that dried fruit intake and fresh fruit intake were associated with a reduced risk of asthma, and alcohol intake frequency was associated with an increased risk of asthma. This study also found that other factors included in this study were not associated with asthma.

## 1 Introduction

As a common chronic disease, asthma is a major health problem worldwide (1). The incidence of asthma has increased rapidly over the past few decades (2–4). The goal of asthma treatment is to minimize both symptoms and the risk of adverse outcomes (5). The increase in asthmatic patients has increased the medical burden worldwide. In Western countries, the financial burden for an individual asthma patient ranges from US$300 to US$1,300 per year (6). Asthma patients in developing countries face both a financial burden and a higher risk of adverse outcomes due to appropriate treatment is deficient. Dietary factors may play an important role in this increase in asthma (7). In adults, the causes of asthma include environmental and lifestyle factors. A previous study has reported the critical role of diet in the development of allergic diseases (8). Dietary factors may be directly related to asthma pathogenesis (9). Knowing whether dietary changes benefit patients with asthma has important implications for both clinicians and patients with asthma. Previous studies have found that alcohol intake (10), fruit intake (11), vegetable intake (12), dairy intake (13), fish intake (14), and meat intake (15) were associated with asthma or asthma symptoms. Mendelian randomization (MR) uses genetic variants as instrumental variables (IVs), which has advantages over other research methods (16). However, there are few MR studies on the causal relationships between dietary factors and asthma. We, therefore, performed this MR analysis to explore the relationships between dietary factors and asthma.

## 2 Methods

The following basic assumptions constitute the premise of MR analysis. First, IVs must be intensely associated with the exposure factor(s). Second, IVs cannot be directly correlated to the outcome. Third, IVs were not related to any potential confounding factors. The GWAS summary-level data used in this study was issued by the IEU open GWAS project. This project, supported by the MRC Integrative Epidemiology Unit (IEU) at the University of Bristol, collated and analyzed GWAS data from UK Biobank, published articles, and FinnGen biobank. This study was exempt from the approval of the Ethical Review Authority because the data used in this study was public, anonymized, and de-identified.

### 2.1 Data sources

Diet-Related Exposure factors used in this study included vegetable intake (Salad/raw vegetable intake and Cooked vegetable intake), meat intake (Processed meat intake, Poultry intake, Beef intake, Non-oily fish intake, Oily fish intake, Pork intake, and Lamb/mutton intake), staple food intake (Bread intake and Cereal intake), beverage intake (Alcoholic drinks per week, Alcohol intake frequency, Tea intake, and Coffee intake), fruit intake(Dried fruit intake and Fresh fruit intake), and another food intake (Cheese intake). These GWAS summary-level data were extracted directly or indirectly from UK Biobank by the IEU open GWAS project. The GWAS summary-level data of asthma was extracted from FinnGen biobank by the IEU open GWAS project. We did not use proxy SNPs when finding SNPs from the outcome, mainly because the FinnGen biobank contained enough SNPs (16,380,176 SNPs in the dataset of asthma). More information about the exposure and outcome datasets is presented in **Table 1** and **Supplementary Table 1**.

**Table 1:** Information of the exposures and outcome datasets.

IEU GWAS id	Exposure or outcome	Identified SNPs	Participants included in analysis	F-statistic
ieu-b-73	Alcoholic drinks per week	33	335394 European-descent individuals	97.217
ukb-b-5779	Alcohol intake frequency	92	462346 European-descent individuals	112.254
ukb-b-6324	Processed meat intake	23	461981 European-descent individuals	39.164
ukb-b-8006	Poultry intake	7	461900 European-descent individuals	24.621
ukb-b-2862	Beef intake	14	461053 European-descent individuals	28.481
ukb-b-17627	Non-oily fish intake	11	460880 European-descent individuals	26.780
ukb-b-2209	Oily fish intake	60	460443 European-descent individuals	37.644
ukb-b-5640	Pork intake	13	460162 European-descent individuals	18.849
ukb-b-14179	Lamb/mutton intake	30	460006 European-descent individuals	19.030
ukb-b-11348	Bread intake	25	452236 European-descent individuals	38.339
ukb-b-1489	Cheese intake	61	451486 European-descent individuals	44.815
ukb-b-8089	Cooked vegetable intake	17	448651 European-descent individuals	20.679
ukb-b-6066	Tea intake	39	447485 European-descent individuals	63.637
ukb-b-3881	Fresh fruit intake	52	446462 European-descent individuals	15.502
ukb-b-15926	Cereal intake	38	441640 European-descent individuals	32.723
ukb-b-1996	Salad / raw vegetable intake	18	435435 European-descent individuals	17.650
ukb-b-5237	Coffee intake	38	428860 European-descent individuals	41.751
ukb-b-16576	Dried fruit intake	39	421764 European-descent individuals	25.412
finn-b-J10_ASTHMA	Asthma	NA	20629 European-descent cases and 135449 European-descent controls	NA

The information of the exposure and outcome datasets. SNPs, Single-nucleotide polymorphisms. IEU, Integrative Epidemiology Unit; GWAS, Genome-Wide Association Studies. N/A, Not applicable.

### 2.2 The selection of IVs

In MR analysis, IVs were utilized as mediators between exposure factors and outcomes to explore the causal relationship between exposure and outcomes. IVs are generally genetic variations, among which Single nucleotide polymorphisms (SNPs) are the most commonly used. SNPs associated with dietary factors were extracted from the IEU open GWAS project (https://gwas.mrcieu.ac.uk/). we screened the SNPs intensely related with exposures at the genome-wide significance level (p < 5×10^–8^), clumping window > 10,000 kb, and the linkage disequilibrium level (r2 < 0.001). More information is shown in **Table 1**. The F statistic was used to ensure the strong association between IVs and exposure, The F statistic greater than 10 was generally considered to meet the requirements of strong association (17).

### 2.3 Statistical analysis

We conducted the inverse variance weighted (IVW) method as the primary method for calculating the causal effect. The IVW model is the method with the strongest ability to detect causation in the two-sample MR analysis (18). We contrasted the consequences of the IVW method with the weighted median and the MR-Egger methods. The weighted median method allows no more than 50% of invalid IVs, and the MR-Egger method allows all IVs to be voided. Therefore, it will be more convincing when the three models are consistent. The heterogeneity of the IVW model was assessed by Cochran’s Q test. Cochran’s Q-test p<0.05 indicated heterogeneity. However, the existence of heterogeneity does not mean that the IVW model is necessarily invalid. The MR-Egger method allows for the existence of non-zero intercepts and was used to detect directional pleiotropy. Leave-one-out analysis was performed to assess whether there was a significant effect on the results after the removal of a single SNP. We use the MR-PRESSO method to detect outliers. Once outliers were found, they will be removed immediately. After removing outliers, the MR analysis will be performed again. All analyses were performed in R software (version 4.2.0) using the TwoSampleMR package (19).

## 3 Results

The causal relationship between dietary factors and asthma were analyzed using 18 different exposure factors. The amounts of SNPs used in this study ranged from 7 to 92. The F-statistics are all greater than 10 (range: 15.502 to 112.254). The number of European-descent individuals included in the exposures ranged from 335,394 to 462,346. The outcome included 20 629 European-descent asthma cases and 135 449 European-descent controls from the FinnGen biobank, and there was little overlap between the populations involved in exposures and outcome. See **Table 1** for more information on exposures and results. The number of SNPs used for different exposures in this study ranged from 7 to 92, and the range after removing outliers was between 5 and 90 (the number of outliers found in different exposures ranged from 0 to 2). See **Table 2** for more information. As shown in **Table 1**, the F statistic (after removing outliers) ranges from 15.502 to 112.254, which indicates that IVs used in our study satisfies the requirements of strong association with exposures.

**Table 2:** The results of Mendelian randomization analyses.

	Exposure	Used SNPs	inverse variance weighted method		Weighted median method		MR-Egger method		Cochrane's Q test		Pleiotropy			MR-PRESSO a							Outliers excluded b										
			OR(95% CI)	P-value	OR(95% CI)	P-value	OR(95% CI)	P-value	Q	P-value	MR-Egger intercept	se	P-value	Raw			Outliers	outlier-corrected			inverse variance weighted method		Weighted median method		MR-Egger method		Cochrane’s Q test		Pleiotropy		
														casual estimate	sd	P-value		casual estimate	sd	P-value	OR(95% CI)	P-value	OR(95% CI)	P-value	OR(95% CI)	P-value	Q	P-value	MR-Egger intercept	se	P-value
**ieu-b-73**	**Alcoholic drinks per week**	**33**	**0.903(0.599-1.359)**	**0.624**	**0.651(0.409-1.036)**	**0.070**	**0.884(0.341-2.292)**	**0.802**	**65.832**	**0.000395**	**0.000386**	**0.00827**	**0.963**	**-0.102**	**0.209**	**0.627**	**rs28712821;rs28929474**	**-0.219**	**0.169**	**0.206**	**0.804(0.577-1.120)**	**0.196**	**0.633(0.402-0.996)**	**0.0484**	**0.730(0.328-1.625)**	**0.448**	**34.783**	**0.251**	**0.00170**	**0.00659**	**0.798**
**ukb-b-5779**	**Alcohol intake frequency**	**92**	**1.173(0.999-1.378)**	**0.0513**	**1.254(1.033-1.521)**	**0.0221**	**1.192(0.722-1.967)**	**0.494**	**174.852**	**3.05E-07**	**-0.00039**	**0.00607**	**0.949**	**0.160**	**0.082**	**0.0544**	**rs28768122;rs11940694**	**0.197**	**0.0763**	**0.0116**	**1.217(1.048-1.413)**	**0.00993**	**1.270(1.0462-1.542)**	**0.0157**	**1.624(1.010-2.610)**	**0.0483**	**139.917**	**0.000464**	**-0.00709**	**0.00566**	**0.213**
**ukb-b-6324**	**Processed meat intake**	**23**	**1.315(0.797-2.168)**	**0.284**	**1.284(0.746-2.208)**	**0.367**	**1.020(0.079-13.254)**	**0.988**	**39.941**	**0.0109**	**0.00384**	**0.0194**	**0.845**	**0.27300**	**0.255**	**0.295**	**rs2029401**	**0.127**	**0.232**	**0.588**	**1.136(0.721-1.790)**	**0.583**	**1.272(0.722-2.241)**	**0.404**	**1.004(0.104-9.709)**	**0.998**	**29.760**	**0.0969**	**0.00188**	**0.0172**	**0.914**
**ukb-b-8006**	**Poultry intake**	**7**	**1.101(0.151-8.051)**	**0.924**	**1.124(0.280-4.508)**	**0.869**	**3.537E-18(4.064E-42-3.078E+06)**	**0.212**	**32.126**	**1.54E-05**	**0.436**	**0.304**	**0.211**	**0.0963**	**1.015**	**0.928**	**rs1051730;rs9997448**	**0.603**	**0.599**	**0.371**	**1.827(0.565-5.906)**	**0.314**	**1.025(0.245-4.290)**	**0.973**	**1.449E-14(6.521e-27-0.0322)**	**0.116**	**5.256**	**0.262**	**0.354**	**0.158**	**0.111**
**ukb-b-2862**	**Beef intake**	**14**	**0.807(0.307-2.124)**	**0.664**	**0.462(0.198-1.080)**	**0.0748**	**0.0556(0.000156-19.864)**	**0.354**	**38.305**	**0.000257**	**0.0339**	**0.0375**	**0.383**	**-0.215**	**0.494**	**0.671**	**rs62169335;rs429358**	**-0.318**	**0.387**	**0.428**	**0.479(0.227-1.012)**	**0.0538**	**0.458(0.194-1.080)**	**0.0745**	**0.0143(0.000152-1.351)**	**0.0971**	**16.712**	**0.117**	**0.0449**	**0.0293**	**0.157**
**ukb-b-17627**	**Non-oily fish intake**	**11**	**0.957(0.302-3.029)**	**0.940**	**0.681(0.243-1.912)**	**0.466**	**0.0187(0.000107-3.267)**	**0.165**	**31.48**	**0.000488**	**0.0488**	**0.0320**	**0.161**	**-0.0440**	**0.588**	**0.942**	**rs56094641**	**-0.433**	**0.554**	**0.455**	**0.648(0.219-1.923)**	**0.435**	**0.430(0.144-1.285)**	**0.131**	**0.0112(0.000155-0.810)**	**0.0737**	**22.006**	**0.00886**	**0.0503**	**0.0264**	**0.093**
**ukb-b-2209**	**Oily fish intake**	**60**	**0.739(0.501-1.091)**	**0.128**	**0.713(0.479-1.062)**	**0.0961**	**0.539(0.103-2.816)**	**0.467**	**161.092**	**2.06E-11**	**0.00469**	**0.0122**	**0.702**	**-0.302**	**0.199**	**0.134**	**rs1421085;rs2952140**	**-0.287**	**0.166**	**0.0885**	**0.751(0.543-1.038)**	**0.0831**	**0.654(0.438- 0.977)**	**0.0379**	**0.219(0.0570-0.842)**	**0.0311**	**102.784**	**0.000193**	**0.0183**	**0.00991**	**0.0702**
**ukb-b-5640**	**Pork intake**	**13**	**1.287(0.429-3.863)**	**0.653**	**0.766(0.258-2.275)**	**0.631**	**0.297(0.000244-361.855)**	**0.744**	**30.325**	**0.00249**	**0.0152**	**0.0371**	**0.690**	**0.252**	**0.561**	**0.661**	**rs12721051;rs2387807**	**0.203**	**0.459**	**0.668**	**1.225(0.498-3.013)**	**0.658**	**0.755(0.247-2.308)**	**0.622**	**1.173(0.000271-5073.120)**	**0.971**	**13.455**	**0.199**	**0.000427**	**0.0416**	**0.992**
**ukb-b-14179**	**Lamb/mutton intake**	**30**	**1.127(0.564-2.251)**	**0.735**	**0.954(0.427-2.132)**	**0.908**	**2.627(0.138-49.982)**	**0.526**	**65.171**	**0.000135**	**-0.00940**	**0.0162**	**0.567**	**0.120**	**0.353**	**0.737**	**rs429358**	**-0.133**	**0.335**	**0.694**	**0.875(0.454-1.687)**	**0.691**	**0.843(0.405-1.755)**	**0.648**	**0.303(0.0134-6.819)**	**0.458**	**52.012**	**0.00382**	**0.0114**	**0.0167**	**0.499**
**ukb-b-11348**	**Bread intake**	**25**	**0.942(0.615-1.442)**	**0.784**	**0.884(0.520-1.501)**	**0.647**	**1.090(0.146-8.164)**	**0.934**	**34.310**	**0.0793**	**-0.00212**	**0.0146**	**0.885**	**-0.0596**	**0.217**	**0.786**	**NA**	**NA**	**NA**	**NA**	**NA**	**NA**	**NA**	**NA**	**NA**	**NA**	**NA**	**NA**	**NA**	**NA**	**NA**
**ukb-b-1489**	**Cheese intake**	**61**	**0.876(0.678-1.132)**	**0.310**	**1.019(0.727-1.427)**	**0.914**	**0.513(0.172-1.533)**	**0.237**	**89.582**	**0.00795**	**0.00919**	**0.00934**	**0.329**	**-0.133**	**0.131**	**0.314**	**NA**	**NA**	**NA**	**NA**	**NA**	**NA**	**NA**	**NA**	**NA**	**NA**	**NA**	**NA**	**NA**	**NA**	**NA**
**ukb-b-8089**	**Cooked vegetable intake**	**17**	**1.230(0.558-2.710)**	**0.607**	**0.987(0.403-2.416)**	**0.977**	**4.737(0.000627-35769.624)**	**0.738**	**29.263**	**0.0222**	**-0.0139**	**0.0469**	**0.770**	**0.207**	**0.403**	**0.614**	**rs1421085**	**-0.0178**	**0.371**	**0.962**	**0.982(0.475-2.031)**	**0.962**	**0.648(0.253-1.654)**	**0.364**	**3.737(0.00134-10436.562)**	**0.750**	**21.594**	**0.119**	**-0.0138**	**0.0416**	**0.745**
**ukb-b-6066**	**Tea intake**	**39**	**0.827(0.621-1.101)**	**0.192**	**0.897(0.641-1.255)**	**0.526**	**0.963(0.512-1.809)**	**0.907**	**63.357**	**0.00606**	**-0.00326**	**0.00614**	**0.598**	**-0.190**	**0.146**	**0.200**	**rs2279844**	**-0.232**	**0.136**	**0.0949**	**0.793(0.608-1.034)**	**0.0865**	**0.847(0.601-1.194)**	**0.344**	**1.053(0.590-1.877)**	**0.863**	**52.559**	**0.0466**	**-0.00615**	**0.00569**	**0.287**
**ukb-b-3881**	**Fresh fruit intake**	**52**	**0.489(0.320-0.748)**	**0.000954**	**0.462(0.255-0.838)**	**0.0110**	**0.971(0.229-4.122)**	**0.968**	**61.804**	**0.143**	**-0.00659**	**0.00678**	**0.336**	**-0.7150**	**0.217**	**0.00175**	**NA**	**NA**	**NA**	**NA**	**NA**	**NA**	**NA**	**NA**	**NA**	**NA**	**NA**	**NA**	**NA**	**NA**	**NA**
**ukb-b-15926**	**Cereal intake**	**38**	**0.617(0.404-0.943)**	**0.0256**	**0.607(0.373-0.987)**	**0.0442**	**0.338(0.0546-2.095)**	**0.252**	**72.903**	**0.000389**	**0.00882**	**0.0133**	**0.510**	**-0.483**	**0.216**	**0.0317**	**rs11940694**	**-0.398**	**0.204**	**0.0586**	**0.671(0.450-1.001)**	**0.0508**	**0.612(0.367-1.021)**	**0.0601**	**0.291(0.0537-1.575)**	**0.161**	**61.489**	**0.00511**	**0.0123**	**0.0123**	**0.325**
**ukb-b-1996**	**Salad / raw vegetable intake**	**18**	**0.810(0.287-2.284)**	**0.691**	**2.264(0.767-6.683)**	**0.139**	**16.837(0.155-1824.858)**	**0.254**	**41.510**	**0.000789**	**-0.0329**	**0.0253**	**0.212**	**-0.211**	**0.529**	**0.695**	**rs10819082**	**0.0260**	**0.486**	**0.958**	**1.026(0.396-2.660)**	**0.957**	**2.276(0.791-6.552)**	**0.127**	**9.368(0.125-703.379)**	**0.326**	**31.464**	**0.0117**	**-0.0242**	**0.0235**	**0.320**
**ukb-b-5237**	**Coffee intake**	**38**	**0.947(0.676-1.328)**	**0.754**	**0.977(0.642-1.486)**	**0.914**	**1.021(0.514-2.031)**	**0.952**	**58.495**	**0.0137**	**-0.00142**	**0.00575**	**0.807**	**-0.0540**	**0.172**	**0.756**	**NA**	**NA**	**NA**	**NA**	**NA**	**NA**	**NA**	**NA**	**NA**	**NA**	**NA**	**NA**	**NA**	**NA**	**NA**
**ukb-b-16576**	**Dried fruit intake**	**39**	**0.521(0.339-0.780)**	**0.00287**	**0.478(0.290-0.788)**	**0.00382**	**0.174(0.0263-1.153)**	**0.0780**	**64.068**	**0.00514**	**0.0137**	**0.0117**	**0.251**	**-0.652**	**0.219**	**0.00498**	**rs11152349**	**-0.729**	**0.202**	**0.000915**	**0.482(0.325-0.717)**	**0.000312**	**0.477(0.284-0.801)**	**0.00510**	**0.231(0.0398-1.343)**	**0.112**	**52.398**	**0.0481**	**0.00923**	**0.0110**	**0.406**

OR, Odds ratio; SNPs, Single-nucleotide polymorphisms; CI, Confidence interval; NA, Not available;

a: The results of MR-PRESSO are presented in the form of beta values, and there is a conversion relationship between beta values and OR, specifically beta=log(OR).

b:We repeated the Mendelian randomization analysis after removing outliers.

**Figure 1:**
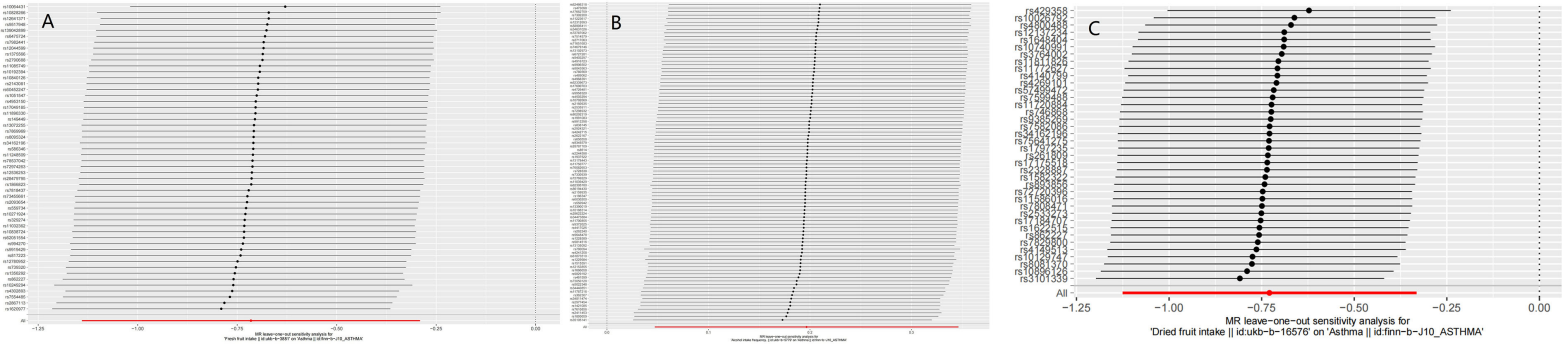
The results of Leave-one-out analyses **(A)** Fresh fruit intake **(B)** Alcohol intake frequency **(C)** Dried fruit intake.

In this study, a total of 3 causalities were identified (p < 0.05 by IVW method). We found that alcohol intake frequency (after removing outliers OR: 1.217; 95% CI: 1.048-1.413; p=0.00993) was related to an increased risk of Asthma. This discovery was further verified by the consequences of the MR-Egger (after removing outliers OR: 1.624; 95% CI: 1.010-2.610; p= 0.0483) and weighted median (after removing outliers OR: 1.270; 95% CI: 1.046-1.542; p=0.0157) model. Fresh fruit intake (OR: 0.489; 95% CI: 0.320-0.748; p=0.000954) and Dried fruit intake (after removing outliers OR: 0.482; 95% CI: 0.325-0.717; p= 0.000312) were discovered as protective factors. And we have reached the same conclusion in the Weighted median model (Fresh fruit intake OR: 0.462; 95% CI: 0.255-0.838; p=0.011; Dried fruit intake OR: 0.477; 95% CI: 0.284-0.801; p=0.0051). However, there were no significant results in the MR-Egger model (P>0.05). Cereal intake showed a positive result before the outliers were not removed, and the positive result disappeared after the outlier was removed (P= 0.0256 *VS* 0.0508). This study also found that Alcoholic drinks per week (OR: 0.903; 95% CI: 0.599-1.359; p=0.624; Outliers excluded: OR:0.804; 95% CI:0.577-1.120; p=0.196), Processed meat intake (OR: 1.315; 95% CI: 0.797-2.168; p=0.284; Outliers excluded: OR:1.136; 95% CI:0.721-1.790; p=0.583), Poultry intake (OR: 1.101; 95% CI: 0.151-8.051; p= 0.924; Outliers excluded: OR:1.827; 95% CI:0.565-5.906; p=0.314), Beef intake (OR: 0.807; 95% CI: 0.307-2.124; p= 0.664; Outliers excluded: OR:0.479; 95% CI:0.227-1.012; p=0.0538), Non-oily fish intake (OR: 0.957; 95% CI: 0.302-3.029; p= 0.940; Outliers excluded: OR:0.648; 95% CI:0.219-1.923; p=0.435), Oily fish intake (OR: 0.739; 95% CI: 0.501-1.091; p= 0.128; Outliers excluded: OR:0.751; 95% CI:0.543-1.038; p=0.0831), Pork intake (OR: 1.287; 95% CI: 0.429-3.863; p= 0.653; Outliers excluded: OR:1.225; 95% CI:0.498-3.013; p=0.658), and Lamb/mutton intake (OR: 1.127; 95% CI: 0.564-2.251; p= 0.735; Outliers excluded: OR:0.875; 95% CI:0.454-1.687; p=0.691), Bread intake(OR: 0.942; 95% CI: 0.615-1.442; p= 0.784; No outliers), Cheese intake(OR: 0.876; 95% CI: 0.678-1.132; p= 0.310; No outliers), Cooked vegetable intake(OR: 1.230; 95% CI: 0.558-2.710; p= 0.607; Outliers excluded: OR:0.982; 95% CI:0.475-2.031; p= 0.962), Tea intake(OR: 0.827; 95% CI: 0.621-1.101; p= 0.192; Outliers excluded: OR:0.793; 95% CI:0.608-1.034; p=0.0865), Salad/raw vegetable intake(OR: 0.810; 95% CI: 0.287-2.284; p= 0.691; Outliers excluded: OR:1.026; 95% CI:0.396-2.660; p=0.957), and Coffee intake(OR: 0.947; 95% CI: 0.676-1.328; p= 0.754; No outliers) were not associated with asthma either before or after exclusion of outliers. More MR analysis results are in **Table 2**. Although heterogeneity was discovered in a considerable number of exposures (Cochrane’s Q test P<0.05), the consequences of the MR-Egger intercept suggested that no directional pleiotropy was discovered (**Table 2**). Leave-one-out analysis indicated that the causalities of the positive results were very robust (**Figure 1**). As shown in **Table 2**, the results of the MR-PRESSO analysis were greatly consistent with the results of the IVW model (causal relationships were only shown in Alcohol intake frequency, Fresh fruit intake, and Dried fruit intake).

## 4 Discussion

The most important finding of this MR analysis is that Alcohol intake frequency, Fresh fruit intake, and Dried fruit intake were associated with asthma. Other findings are also noteworthy. First of all, a causal relationship between Cereal intake and asthma cannot be completely ruled out. In our analysis, if there were any outliers, we repeated the MR analysis and used the new results of the IVW model as a basis for determining whether there was a causal relationship. When we analyzed whether there was a causal relationship between grain intake and asthma, we found no causal relationship after excluding outliers (OR: 0.671; 95% CI: 0.450-1.001; p=0.0508). And a causal relationship was shown in the IVW model (OR: 0.617; 95% CI: 0.404-0.943; p= 0.0256) and the weighted median model (OR: 0.607; 95% CI: 0.373-0.987; p= 0.0442) before excluding outliers. We, therefore, think that the 95% confidence intervals and p-values at critical value have limited convincing power. Secondly, Alcoholic drinks per week, meat intake (Processed meat intake, Poultry intake, Beef intake, Non-oily fish intake, Oily fish intake, Pork intake, and Lamb/mutton intake), Bread intake, Cheese intake, Cooked vegetable intake, Tea intake, Salad/raw vegetable intake, and Coffee intake were not associated with asthma. To our knowledge, there have been many MR studies on the risk or protective factors of asthma (20, 21). However, there are few studies involving meat intake, staple food intake, fruit and vegetable intake, and beverage intake. Asthma imposes a heavy economic burden on the world every year. Asthma and asthma complications also seriously affect the quality of life of patients with asthma. The conclusions of our study can help clinicians to improve their health education for patients with asthma, and encourage patients with asthma to change their eating habits (such as reducing the frequency of alcohol intake and increasing fruit intake). For those at high risk for asthma, adjusting dietary habits also reduces the risk of developing asthma. Therefore, this study has important implications for deepening the understanding of the risk and protective factors of asthma.

Alcohol can affect the human immune system (22, 23). Numerous studies have shown that heavy drinking is associated with higher immunoglobulin E (IgE) levels (24–26). Alcohol consumption is also associated with the development of allergic diseases, such as allergic rhinitis and atopic dermatitis (27, 28). A prospective study found a U-shaped association between alcohol consumption and the risk of asthma, with moderate alcohol consumption having the lowest asthma risk (10). However, Mendelian randomization analysis did not find a causal relationship between alcohol intake and asthma (29, 30). Unlike observational studies, MR studies using genetic variations (primarily SNPs) as instrumental variables are immune to confounding factors and reverse causality. This study found no causality between Alcoholic drinks per week and asthma, however, there was a causal relationship between the frequency of alcohol intake and asthma. We believe that the possible reasons for the differences between MR studies and observational studies in the relationship between alcohol intake and asthma are as follows: First, although possible confounding factors have been adjusted, observational studies may still be affected by other confounding factors. Second, there is indeed a U-shaped association between alcohol intake and asthma, which was not detected by the MR study. Therefore, more observational studies and more ingenious MR studies are needed in the future to further reveal the relationship between alcohol intake and asthma. Our study indicated that there may indeed be a causal relationship between alcohol intake and asthma, and this causal relationship is more about the frequency of alcohol intake than the amount of alcohol intake.

The airway is particularly vulnerable to oxidative damage. In experiments, oxidants can induce many symptoms of asthma by inducing the release of pro-inflammatory mediators including cytokines and chemokines (31). Vegetables and fruits contain many antioxidants. Studies in children showed that asthma symptoms were inversely correlated with fish, vegetable, and fruit intake (14, 32). Several studies in adults have linked high fruit and vegetable intake to a lower risk of asthma (33–36). However, not all studies have shown an association between vegetable intake and asthma. However, not all studies have shown an association between vegetable intake and asthma. A study found no association between vegetable intake and asthma (37). This study used the MR analysis method to find a causal relationship between fruit intake and asthma, but not vegetable intake. There have been many studies on the relationship between fish intake and asthma (38–41). Most of these studies supported an association between fish intake and a reduced risk of asthma (38–41). A Japanese study found that the risk of childhood asthma increased with the frequency of fish intake (42). However, our study showed no causal relationship between fish intake (including Oily fish intake and Non-oily fish intake) and asthma. This study provides powerful new evidence for insights into the relationship between fish intake and asthma. A study from Singapore suggested that a diet rich in meats may increase the risk of cough with phlegm (15). A study from Australia found a positive association between meat/cheese intake, poultry/seafood intake and asthma or hayfever (43). However, this study found no causal relationship between either meat intake or poultry intake and asthma. A study from South Korea found coffee intake had a protective effect on asthma (44). A prospective cohort study from the UK Biobank has found that coffee and tea intake may be protective against asthma (45). This study found coffee and tea intake were not associated with asthma. We must correctly understand the relationship between MR and randomized controlled trials (RCTs). On the one hand, MR can effectively overcome the bias caused by confounding (16). on the other hand, As a powerful supplement to randomized controlled trials, MR is not a substitute for RCTs. Therefore, this conclusion must be viewed with caution.

The mechanisms by which dietary factors affect asthma are not fully understood. Antioxidants and lipids may play an important role (46). Another possible pathway is through the gut microbiome. The intake of different foods can affect the composition of bacteria in the gut, which will affect the metabolism of nutrients (47, 48). A link between changes in the gut microbiome and improvements in airway hyperresponsiveness was found in a mouse model (49).

Although this MR analysis suggested associations between Fresh fruit intake, Dried fruit intake, and alcohol intake and asthma, the MR analysis should be interpreted with more caution. First, the causality found by the MR analysis reflected the effects of long-term exposure to associated factors. Therefore, short-term exposures may not be of clinical significance. Second, another notable problem is that MR cannot distinguish causal relationships between different periods. For example, an MR study found a causality between vitamin D and multiple sclerosis (50). However, this effect was only present in childhood or earlier (51). Third, Univariate MR analyses revealed only overall effects between exposures and outcomes, not direct effects between them. There can be extremely complex mechanisms between exposures and outcomes.

This study has some strengths and limitations. MR uses genetic variation as IVs to infer the causality, which can effectively overcome the bias caused by reverse causality and confounding (16). In order to ensure the accuracy of MR analysis, we performed sensitivity and pleiotropic analysis. We used European populations from different countries in exposures and outcome to avoid unnecessary bias. Of course, the limitations of this study cannot be ignored. First, the F-statistics indicated that the IVs used in this study satisfied the requirement of strong associations with exposure (F-statistics>10). however, a considerable part of the F-statistics is lower than 100, so this may affect the accuracy of the consequences. Second, we cannot further subdivide different types of dietary intake, nor can we distinguish the effects of different dietary combinations. Third, we were unable to conduct a sex-stratified analysis due to the lack of summary-level GWAS data for different sexes.

## 5 Conclusion

This study found that dried fruit intake and fresh fruit intake were associated with a reduced risk of asthma, and alcohol intake frequency was associated with an increased risk of asthma. This study also found that alcoholic drinks per week, processed meat intake, poultry intake, beef intake, non-oily fish intake, oily fish intake, pork intake, lamb/mutton intake, bread intake, cheese intake, cooked vegetable intake, tea intake, cereal intake, salad/raw vegetable intake, and coffee intake were not associated with asthma.

## Data availability statement

Publicly available datasets were analyzed in this study. This data can be found here: All GWAS data used in this study are available in the IEU open GWAS project (https://gwas.mrcieu.ac.uk/).

## Ethics statement

Ethical review and approval was not required for the study on human participants in accordance with the local legislation and institutional requirements. Written informed consent was not provided because All GWAS data used in this study are available in the IEU open GWAS project (https://gwas.mrcieu.ac.uk/). This study was exempt from the approval of the Ethical Review Authority because the data used in this study was public, anonymized, and de-identified.

## Author contributions

The study was designed by WY and BH. Statistical analyses were performed by WY, LH, MZ, SS, FW and YY. The manuscript was written by WY and YY. All authors contributed to the interpretation of data and commented on the manuscript. All authors read and approved the manuscript. All authors contributed to the article and approved the submitted version.

## References

[1] Asher MI Montefort S Björkstén B Lai CK Strachan DP Weiland SK. Worldwide time trends in the prevalence of symptoms of asthma, allergic rhinoconjunctivitis, and eczema in childhood: ISAAC phases one and three repeat multicountry cross-sectional surveys. Lancet (2006) 368(9537):733–43. doi: 10.1016/S0140-6736(06)69283-0 16935684

[2] Moorman JE Akinbami LJ Bailey CM Zahran HS King ME Johnson CA. National surveillance of asthma: United states, 2001-2010. In: Vital & health statistics. series 3, analytical and epidemiological studies (2012) (Hyattsville, Maryland: National Center for Health Statistics), vol. 2012. . p. 1–58 Available at: https://www.cdc.gov/nchs/data/series/sr_03/sr03_035.pdf. 24252609

[3] Akinbami LJ Moorman JE Bailey C Zahran HS King M Johnson CA. Trends in asthma prevalence, health care use, and mortality in the united states, 2001-2010. In: NCHS data brief (2012(94) (Hyattsville, Maryland: National Center for Health Statistics). p. 1–8. 22617340

[4] Stern J Pier J Litonjua AA. Asthma epidemiology and risk factors. Semin immunopathology. (2020) 42(1):5–15. doi: 10.1007/s00281-020-00785-1 32020334

[5] Papi A Brightling C Pedersen SE Reddel HK. Asthma. Lancet (2018) 391(10122):783–800. doi: 10.1016/S0140-6736(17)33311-1 29273246

[6] Braman SS. The global burden of asthma. Chest (2006) 130(1 Suppl):4s–12s. doi: 10.1378/chest.130.1_suppl.4S 16840363

[7] Seaton A Godden DJ Brown K. Increase in asthma: a more toxic environment or a more susceptible population? Thorax (1994) 49(2):171–4. doi: 10.1136/thx.49.2.171 PMC474339 8128408

[8] Julia V Macia L Dombrowicz D. The impact of diet on asthma and allergic diseases. Nat Rev Immunol (2015) 15(5):308–22. doi: 10.1038/nri3830 25907459

[9] Guo CH Liu PJ Lin KP Chen PC. Nutritional supplement therapy improves oxidative stress, immune response, pulmonary function, and quality of life in allergic asthma patients: an open-label pilot study. Altern Med Rev J Clin therapeutic. (2012) 17(1):42–56. 22502622

[10] Lieberoth S Backer V Kyvik KO Skadhauge LR Tolstrup JS Grønbæk M. Intake of alcohol and risk of adult-onset asthma. Respir Med (2012) 106(2):184–8. doi: 10.1016/j.rmed.2011.11.004 22129491

[11] Butland BK Strachan DP Anderson HR. Fresh fruit intake and asthma symptoms in young British adults: confounding or effect modification by smoking? Eur Respir J (1999) 13(4):744–50. doi: 10.1034/j.1399-3003.1999.13d08.x 10362034

[12] Seyedrezazadeh E Moghaddam MP Ansarin K Vafa MR Sharma S Kolahdooz F. Fruit and vegetable intake and risk of wheezing and asthma: a systematic review and meta-analysis. Nutr Rev (2014) 72(7):411–28. doi: 10.1111/nure.12121 24947126

[13] Woods RK Walters EH Raven JM Wolfe R Ireland PD Thien FC. Food and nutrient intakes and asthma risk in young adults. Am J Clin Nutr (2003) 78(3):414–21. doi: 10.1093/ajcn/78.3.414 12936923

[14] Chatzi L Torrent M Romieu I Garcia-Esteban R Ferrer C Vioque J. Diet, wheeze, and atopy in school children in menorca, Spain. Pediatr Allergy Immunol (2007) 18(6):480–5. doi: 10.1111/j.1399-3038.2007.00596.x 17680906

[15] Butler LM Koh WP Lee HP Tseng M Yu MC London SJ. Prospective study of dietary patterns and persistent cough with phlegm among Chinese singaporeans. Am J Respir Crit Care Med (2006) 173(3):264–70. doi: 10.1164/rccm.200506-901OC PMC1447591 16239624

[16] Boyko EJ. Observational research–opportunities and limitations. J Diabetes its complications. (2013) 27(6):642–8. doi: 10.1016/j.jdiacomp.2013.07.007 PMC3818421 24055326

[17] Staiger DO Stock JH. Instrumental variables regression with weak instruments. Mass., USA: National Bureau of Economic Research Cambridge (1994).

[18] Hartwig FP Davey Smith G Bowden J. Robust inference in summary data mendelian randomization *via* the zero modal pleiotropy assumption. Int J Epidemiol. (2017) 46(6):1985–98. doi: 10.1093/ije/dyx102 PMC5837715 29040600

[19] Hemani G Zheng J Elsworth B Wade KH Haberland V Baird D. The MR-base platform supports systematic causal inference across the human phenome. Elife (2018) 7, e34408. doi: 10.7554/eLife.34408 29846171 PMC5976434

[20] Mikkelsen H Landt EM Benn M Nordestgaard BG Dahl M. Causal risk factors for asthma in mendelian randomization studies: A systematic review and meta-analysis. Clin Trans Allergy (2022) 12(11):e12207. doi: 10.1002/clt2.12207 PMC9640961 36434743

[21] Skaaby T Kilpeläinen TO Mahendran Y Huang LO Sallis H Thuesen BH. Association of milk intake with hay fever, asthma, and lung function: a mendelian randomization analysis. Eur J Epidemiol. (2022) 37(7):713–22. doi: 10.1007/s10654-021-00826-5 PMC7614013 34978666

[22] Pasala S Barr T Messaoudi I. Impact of alcohol abuse on the adaptive immune system. Alcohol Res Curr Rev (2015) 37(2):185–97. 10.35946/arcr.v37.2.04 PMC4590616 26695744

[23] Szabo G Saha B. Alcohol's effect on host defense. Alcohol Res Curr Rev (2015) 37(2):159–70. 10.35946/arcr.v37.2.01 PMC4590613 26695755

[24] Domínguez-Santalla MJ Vidal C Viñuela J Pérez LF González-Quintela A. Increased serum IgE in alcoholics: relationship with Th1/Th2 cytokine production by stimulated blood mononuclear cells. Alcoholism Clin Exp Res (2001) 25(8):1198–205. doi: 10.1111/j.1530-0277.2001.tb02336.x 11505051

[25] González-Quintela A Vidal C Gude F Tomé S Lojo S Lorenzo MJ. Increased serum IgE in alcohol abusers. Clin Exp Allergy (1995) 25(8):756–64. doi: 10.1111/j.1365-2222.1995.tb00014.x 7584688

[26] González-Quintela A Vidal C Lojo S Pérez LF Otero-Antón E Gude F. Serum cytokines and increased total serum IgE in alcoholics. Ann allergy Asthma Immunol (1999) 83(1):61–7. doi: 10.1016/S1081-1206(10)63514-4 10437818

[27] Bendtsen P Grønbaek M Kjaer SK Munk C Linneberg A Tolstrup JS. Alcohol consumption and the risk of self-reported perennial and seasonal allergic rhinitis in young adult women in a population-based cohort study. Clin Exp Allergy (2008) 38(7):1179–85. doi: 10.1111/j.1365-2222.2008.02945.x 18294256

[28] Linneberg A Petersen J Grønbaek M Benn CS. Alcohol during pregnancy and atopic dermatitis in the offspring. Clin Exp Allergy (2004) 34(11):1678–83. doi: 10.1111/j.1365-2222.2004.02101.x 15544590

[29] Skaaby T Kilpeläinen TO Taylor AE Mahendran Y Wong A Ahluwalia TS. Association of alcohol consumption with allergic disease and asthma: a multi-centre mendelian randomization analysis. Addict (Abingdon England). (2019) 114(2):216–25. doi: 10.1111/add.14438 PMC7613132 30209858

[30] Lomholt FK Nielsen SF Nordestgaard BG. High alcohol consumption causes high IgE levels but not high risk of allergic disease. J Allergy Clin Immunol (2016) 138(5):1404–1413.e1413. doi: 10.1016/j.jaci.2016.05.022 27464961

[31] Caramori G Papi A. Oxidants and asthma. Thorax (2004) 59(2):170–3. doi: 10.1136/thorax.2002.002477 PMC1746949 14760161

[32] Chatzi L Apostolaki G Bibakis I Skypala I Bibaki-Liakou V Tzanakis N. Protective effect of fruits, vegetables and the Mediterranean diet on asthma and allergies among children in Crete. Thorax (2007) 62(8):677–83. doi: 10.1136/thx.2006.069419 PMC2117278 17412780

[33] Patel BD Welch AA Bingham SA Luben RN Day NE Khaw KT. Dietary antioxidants and asthma in adults. Thorax (2006) 61(5):388–93. doi: 10.1136/thx.2004.024935 PMC2111195 16467075

[34] Shaheen SO Sterne JA Thompson RL Songhurst CE Margetts BM Burney PG. Dietary antioxidants and asthma in adults: population-based case-control study. Am J Respir Crit Care Med (2001) 164(10 Pt 1):1823–8. doi: 10.1164/ajrccm.164.10.2104061 11734430

[35] Romieu I Varraso R Avenel V Leynaert B Kauffmann F Clavel-Chapelon F. Fruit and vegetable intakes and asthma in the E3N study. Thorax (2006) 61(3):209–15. doi: 10.1136/thx.2004.039123 PMC1974844 16396945

[36] Garcia V Arts IC Sterne JA Thompson RL Shaheen SO. Dietary intake of flavonoids and asthma in adults. Eur Respir J (2005) 26(3):449–52. doi: 10.1183/09031936.05.00142104 16135726

[37] Hijazi N Abalkhail B Seaton A. Diet and childhood asthma in a society in transition: a study in urban and rural Saudi Arabia. Thorax (2000) 55(9):775–9. doi: 10.1136/thorax.55.9.775 PMC1745853 10950897

[38] Laerum BN Wentzel-Larsen T Gulsvik A Omenaas E Gíslason T Janson C. Relationship of fish and cod oil intake with adult asthma. Clin Exp Allergy J Br Soc Allergy Clin Immunol (2007) 37(11):1616–23. doi: 10.1111/j.1365-2222.2007.02821.x 17877766

[39] Miyamoto S Miyake Y Sasaki S Tanaka K Ohya Y Matsunaga I. Fat and fish intake and asthma in Japanese women: baseline data from the Osaka maternal and child health study. Int J tuberculosis Lung disease. (2007) 11(1):103–9. 17217138

[40] Hodge L Salome CM Peat JK Haby MM Xuan W Woolcock AJ. Consumption of oily fish and childhood asthma risk. Med J Australia. (1996) 164(3):137–40. doi: 10.5694/j.1326-5377.1996.tb122010.x 8628130

[41] Yang H Xun P He K. Fish and fish oil intake in relation to risk of asthma: a systematic review and meta-analysis. PloS One (2013) 8(11):e80048. doi: 10.1371/journal.pone.0080048 24265794 PMC3827145

[42] Takemura Y Sakurai Y Honjo S Tokimatsu A Gibo M Hara T. The relationship between fish intake and the prevalence of asthma: the tokorozawa childhood asthma and pollinosis study. Prev Med (2002) 34(2):221–5. doi: 10.1006/pmed.2001.0978 11817918

[43] Hooper R Heinrich J Omenaas E Sausenthaler S Garcia-Larsen V Bakolis I. Dietary patterns and risk of asthma: results from three countries in European community respiratory health survey-II. Br J Nutr (2010) 103(9):1354–65. doi: 10.1017/S0007114509993266 19995472

[44] Wee JH Yoo DM Byun SH Song CM Lee HJ Park B. Analysis of the relationship between asthma and Coffee/Green Tea/Soda intake. Int J Environ Res Public Health (2020) 17(20):7471. doi: 10.3390/ijerph17207471 33066553 PMC7602133

[45] Lin F Zhu Y Liang H Li D Jing D Liu H. Association of coffee and tea consumption with the risk of asthma: A prospective cohort study from the UK biobank. Nutrients (2022) 14(19):4039. doi: 10.3390/nu14194039 36235690 PMC9572944

[46] Devereux G Seaton A. Diet as a risk factor for atopy and asthma. J Allergy Clin Immunol (2005) 115(6):1109–17; quiz 1118. doi: 10.1016/j.jaci.2004.12.1139 15940119

[47] Kau AL Ahern PP Griffin NW Goodman AL Gordon JI. Human nutrition, the gut microbiome and the immune system. Nature (2011) 474(7351):327–36. doi: 10.1038/nature10213 PMC3298082 21677749

[48] Muegge BD Kuczynski J Knights D Clemente JC González A Fontana L. Diet drives convergence in gut microbiome functions across mammalian phylogeny and within humans. Sci (New York N.Y.). (2011) 332(6032):970–4. doi: 10.1126/science.1198719 PMC3303602 21596990

[49] Trompette A Gollwitzer ES Yadava K Sichelstiel AK Sprenger N Ngom-Bru C. Gut microbiota metabolism of dietary fiber influences allergic airway disease and hematopoiesis. Nat Med (2014) 20(2):159–66. doi: 10.1038/nm.3444 24390308

[50] Mokry LE Ross S Ahmad OS Forgetta V Smith GD Goltzman D. Vitamin d and risk of multiple sclerosis: A mendelian randomization study. PloS Med (2015) 12(8):e1001866. doi: 10.1371/journal.pmed.1001866 26305103 PMC4549308

[51] Chaudhuri A. Why we should offer routine vitamin d supplementation in pregnancy and childhood to prevent multiple sclerosis. Med hypotheses. (2005) 64(3):608–18. doi: 10.1016/j.mehy.2004.06.022 15617877

